# Multiplayer social dilemmas in completely mixed populations and networks of mixing communities

**DOI:** 10.1007/s00285-026-02464-5

**Published:** 2026-10-01

**Authors:** Diogo L. Pires, Mark Broom

**Affiliations:** 1 Copenhagen Center for Social Data Science (SODAS), University of Copenhagen, Copenhagen, Denmark; 2 Department of Mathematics, City St George’s, University of London, London, UK

**Keywords:** Cooperation Population structure, Evolutionary game theory, Social behaviour, 91A06, 91A22, 91B18, 92D25

## Abstract

Human and non-human animals often form highly structured social systems in which individuals interact preferentially within specific groups, shaped by habitat structure, movement patterns, or social preferences. We consider a model that captures the interplay between social structure and emerging behaviour. Individuals are probabilistically distributed across the nodes of a spatial network representing interaction sites such as resource patches, territorial areas, or social hubs. Individuals present at the same node interact through a multiplayer game, so group structure is not imposed but instead emerges from movement under spatial constraints. As a benchmark, we analyse the completely mixed population, a null model in which individuals are indistinguishable, analogous to the classical well-mixed assumption, and further have no preference for particular network nodes. We derive exact expressions for fundamental properties of this population under arbitrary payoff functions, including the group size distribution, fitness functions, and fixation probabilities. We consider five multiplayer social dilemmas capturing the tension between individual and collective interests, such as those arising in public goods production, collective defence, and communal resource use. For these dilemmas, we derive analytical conditions for the evolution of cooperation in completely mixed populations. Using these as a reference, we reassess the effects of community structure. We show that asymptotically isolated communities expand the parameter range favouring cooperation, particularly under low population density. Finally, we explore intermediate regimes between completely mixed populations and isolated community structure, demonstrating that this expansion of the cooperative region remains robust even under higher levels of mixing between adjacent communities.

## Introduction

The success of social groups, in both humans and non-human animals, often relies on sustained collective behaviour among their members. However, groups frequently face situations where collective benefits can only be achieved if some individuals incur personal costs, creating inherent social dilemmas, often involving multiple individuals (Hamburger 1973; Fox and Guyer 1978; Dawes 1980; Kollock 1998). Similarly to pairwise social dilemmas, multiplayer dilemmas embody collective action problems, where there is a conflict between the social and individual interests, typically framed as a choice between cooperation and defection. Such dilemmas occur widely across species. Public goods dilemmas (Pacheco et al. 2009; Souza et al. 2009; Santos et al. 2015), for example, involve the production of shared benefits, where individuals pay personal costs such as time, energy, predation risk, or access to personal resources, to provide benefits like group safety, hunting success, or the construction and maintenance of communal shelters. Alternatively, common goods dilemmas involve the consumption of existing finite resources subject to depletion (Ostrom 1990; Pires et al. 2025), such as foraging or hunting patches and water sources. Scenarios represented by these dilemmas are found across all levels of biological complexity, from microorganisms (Dormann et al. 2000; Crespi 2001; Kaiser 2004; Gore et al. 2009) to human societies (Hardin 1968; Van Vugt et al. 1996; Skyrms 1996).

Social dilemmas have been extensively modelled using evolutionary game theory, where a wide range of variations have been previously considered in the literature, some of which categorised by Broom et al. (2019). Beyond their theoretical and biological applications, these models have been applied to practical challenges in diverse domains, such as AI monitoring (Alalawi et al. 2026), disease evolution and spread (Morison et al. 2025), environmental governance (Couto et al. 2020), and healthcare investment (Alalawi et al. 2020).

The behaviour adopted by humans and non-human animals facing such dilemmas is strongly influenced by the structure of their social interactions. However, models of evolutionary games often assume well-mixed populations due to the tractability this confers to them. Two examples of this are the extensively used replicator equation (Taylor and Jonker 1978; Hofbauer and Sigmund 1998), and frequency-dependent Moran process (Nowak et al. 2004; Taylor et al. 2004; Pires and Broom 2022). In this context, interactions between individuals are equally likely and therefore individuals are indistinguishable. Furthermore, despite the simplistic assumptions behind them, the results obtained in well-mixed populations are extremely useful as a benchmark for the analysis of evolutionary outcomes on structured populations. However, whereas the well-mixed assumption determines a unique distribution of groups in a population when only pairwise games are considered, multiplayer interactions lead to a wider range of possibilities where individuals are indistinguishable as the distribution of group sizes is unconstrained. For instance, we could have a well-mixed population with all pairwise games, one with all three player games, or one with an equal number of each. We thus propose to develop appropriate benchmarks against which results obtained in structured populations with multiplayer interactions may be compared.

Structured multiplayer interactions have been formulated resorting to different modelling assumptions. In the first accounts, each individual in a pairwise evolutionary graph was determined to interact in a group with all their neighbours simultaneously, as well as to partake in the groups assembled by their neighbours (Santos et al. 2008). Under this account, there is a higher-order interaction network emerging from the underlying evolutionary graph, whose topological features are not fully clear based on the understanding of the original graph. It thus became necessary to develop a more transparent way to describe higher-order interactions in structured populations interacting via multiplayer games. Evolutionary set theory proposed that each individual may be part of several interaction sets which can change dynamically (Tarnita et al. 2009). Similarly, higher-order interactions have been represented by bipartite graphs with a set of nodes for the individuals and another set of nodes denoting potentially interacting groups (Gómez-Gardeñes et al. 2011a, b; Peña and Rochat 2012). These have shown that accounting for interacting groups in a different way may lead to fundamentally different results, even when the corresponding evolutionary graph is the same (Perc et al. 2013). More recently, evolutionary dynamics on hypergraphs have been proposed, where each hyperlink represents an interacting group and selection occurs within them (Alvarez-Rodriguez et al. 2021). These have been used to explain radical behaviour in group decision processes (Civilini et al. 2021), as well as explosive cooperation in multiplayer versions of the prisoner’s dilemma (Civilini et al. 2024). See (Majhi et al. 2022) for a recent more general review of dynamics on higher-order networks.

We consider an alternative framework originally proposed by Broom and Rychtář (2012), where higher-order interactions in the form of multiplayer games emerge from encounters of individuals on networks. Various movement models and their impact on the evolution of cooperative behaviour have been explored within this framework, an overview of which is provided by Broom et al. (2021). These have included completely independent movement between individuals (Broom and Rychtář 2012; Bruni et al. 2014; Broom et al. 2015; Pattni et al. 2017; Schimit et al. 2019, 2022; Pires and Broom 2024), movement contingent on previous interactions (named history-dependent movement) (Pattni et al. 2018; Erovenko et al. 2019; Pires et al. 2023; Erovenko and Broom 2024), and coordinated movement between individuals (row-dependent) (Broom et al. 2020; Haq et al. 2024, 2025). The completely independent movement of individuals has been studied resorting to the territorial raider model in the form introduced in the next section of this paper. In the territorial raider model, each individual has a designated home node, and can either be present there or in one of the adjacent nodes, depending only on a single parameter: their home fidelity *h*. The model is inspired from what is found in animal behaviour (Ketterson and Nolan 1990; Woodroffe et al. 1997; Woodroffe and Ginsberg 1999) and some scales of human mobility (Alessandretti et al. 2020). This model has proven to be useful due to its flexibility, allowing for the study of local movement in small networks (Broom et al. 2015), complex networks (Schimit et al. 2019, 2022), community-structured populations (Pattni et al. 2017; Pires and Broom 2024), as well as working as a base for the study of coordinated herding behaviour (Haq et al. 2024, 2025). Despite the limited prospects for the evolution of cooperation observed under local independent movement (Broom et al. 2015; Pattni et al. 2017; Schimit et al. 2019, 2022), the community structure emerging from overlapping home nodes was proven to sustain it under any network for a set of 10 social dilemmas (Pires and Broom 2024).

In this paper, we will start from the territorial raider model to explore the concept of completely mixed population. A completely mixed population is one where besides all individuals being indistinguishable, all places on the network are indistinguishable as well (Broom and Rychtář 2012). This means that all individuals have the same independent probability of being on any of the places of the network. We start by obtaining this setting from the territorial raider model and deriving characteristics such as group size distribution and fitness. We will analyse fixation processes and obtain simple expressions for the fixation probability of a single mutant on those populations. We will then apply these expressions to five different multiplayer social dilemmas previously analysed and obtain conditions for the evolution of cooperation in completely mixed populations. We argue that these results should be used as a benchmark to which the evolution cooperation under multiplayer interactions should be compared. To show this, we compare the results obtained under completely mixed populations with the rules previously obtained for the evolution of cooperation under community structure (Pires and Broom 2024). Finally, we extend our analysis of the five multiplayer social dilemmas for broader home fidelity values and network topologies in the territorial raider model. We finish by summarising these results and discussing them in light of the remaining literature on multiplayer social dilemmas.

## Modelling evolutionary multiplayer social dilemmas on networks

In this section, we introduce a model for the study of structured multiplayer interactions, with a particular focus on social dilemmas in populations distributed over places. Our main interest lies in deriving the properties of the completely mixed population, defined as one in which individuals are indistinguishable, analogous to the classical well-mixed assumption, and further exhibit no preference for particular network nodes, such that all individuals have the same probability of occupying any available place. We chose to derive the population through the territorial raider model, given that the definition explicitly involves the distribution of individuals over places, and in order to facilitate comparison with the previous extensive results in related studies. In particular, this model is also interesting due to its simplicity as a fully independent movement model, in which individual movement is independent of both previous positions and other individuals, and because of its ability to describe local interactions observed in animal behaviour (Ketterson and Nolan 1990; Woodroffe et al. 1997; Woodroffe and Ginsberg 1999) and at certain scales of human mobility (Alessandretti et al. 2020). We introduce the model here in its full generality, albeit succinctly, since it constitutes both the starting and ending point of this work. The Python implementation of the territorial raider model is publicly available in Pires (2026). For further details, see (Pattni et al. 2017; Pires and Broom 2024) and the original formulation in Broom and Rychtář (2012).

We consider a population composed of *N* individuals $I_n=I_1,...,I_N$. Individuals are positioned on a spatial network with *M* places $P_m=P_1,...,P_M$, with a set of edges connecting them. The probability that an individual $I_n$ is in place $P_m$ is generally defined by a value $p_{nm}$ in a fully independent model. We assume that $p_{nm}$ is time-independent. This can be interpreted as the stationary distribution of an underlying mobility process when movement occurs on a much faster timescale than the evolutionary dynamics. More generally, however, $p_{nm}$ may simply represent a fixed occupancy distribution over locations and does not require the specification of an explicit mobility dynamics. For any set of independent probability distributions $p_{nm}$, a group of individuals $\mathcal {G}$ has probability $\chi (m,\mathcal {G})$ of meeting in node $P_m$, given by:

1 $$\begin{aligned} \chi (m,\mathcal {G})= \prod _{i \in \mathcal {G}} p_{im} \prod _{j \notin \mathcal {G}} (1-p_{jm}). \end{aligned}$$

The fitness of each individual $I_n$ can then be obtained through the weighted average, denoted as $\pi _n$, of the payoffs $R_{n,m,\mathcal {G}}$ received in each place $P_m$ and each group composition $\mathcal {G}$ they can be in. We further introduce *w*, the intensity of selection, which measures the extension to which the outcomes of the game contribute to the fitness of individuals (Nowak et al. 2004):

2 $$\begin{aligned} F_n=1-w + w\pi _n=1-w + w \sum _{m} \sum _{\mathcal {G}: n\in \mathcal {G}} \chi (m,\mathcal {G}) R_{n,m,\mathcal {G}}. \end{aligned}$$

We will consider the fully independent movement model previously introduced as the territorial raider model (Pattni et al. 2017; Pires and Broom 2024), under which each node of the network is home to *Q* individuals. The value *Q* has been previously treated as the community size (Pires and Broom 2024), subpopulation size (Pattni et al. 2017) or, in similar models, the deme size (Hauert and Imhof 2012). In this paper, we will treat *Q* more generally as the *density* of individuals in the network. We denote *h* as the home fidelity parameter, and $d_n$ as the degree of the home node of individual $I_n$. We thus define the set of probability distributions $p_{nm}$ based on considering that each individual $I_n$ has a probability $h/(h+d_n)$ of being in their home node, $1/(h+d_n)$ of being in any of the adjacent nodes, and 0 of being in any other place in the network.

We consider a subset of the multiplayer social dilemmas previously summarised (Broom et al. 2019). Individuals have two strategies available to them: to cooperate, denoted C, or to defect, denoted D. In these dilemmas, payoffs can be represented as $R_{n,m,\mathcal {G}}\equiv R^C_{c,d}$ ($\equiv R^D_{c,d}$) when the focal individual $I_n$ is a cooperator (defector), as they are determined by the type of the focal individual and the total number of cooperators *c*, and defectors *d* in their current group. We present the payoffs received under each social dilemma in Table 1, where *V* represents the value of the reward shared, and *K* the cost paid by individuals in the group. In public goods dilemmas, cooperation involves the production of a reward *V* at a cost *K*, which is consumed by individuals within the group. In contrast, commons dilemmas typically represent scenarios with pre-existing resources, where cooperation can involve, among other things, the sustainable consumption of the resources. In the HD dilemma, the only commons dilemma we study here, cooperators evenly share the reward *V*, while defectors attempt to consume it entirely, either winning it occasionally or losing it to other defectors while incurring a cost *K*.

**Table 1:** Payoffs obtained by a focal cooperator $R^C_{c,d}$ or a focal defector $R^D_{c,d}$ in a group with total *c* cooperators and *d* defectors playing a social dilemma

Multiplayer Game	$R^C_{c,d}$	$R^D_{c,d}$
Charitable Prisoner’s Dilemma (CPD) (Broom et al. 2015)	$${\left\{ \begin{array}{ll} \frac{c-1}{c+d-1}V-K & c>1 \\ -K & c=1 \end{array}\right. } $$	$${\left\{ \begin{array}{ll} \frac{c}{c+d-1}V & c>0 \\ 0 & c=0 \end{array}\right. } $$
Prisoner’s Dilemma (PD) (Hamburger 1973)	$\dfrac{c}{c+d}V -K$	$\dfrac{c}{c+d}V$
Volunteer’s Dilemma (VD) (Diekmann 1985)	V-K	$${\left\{ \begin{array}{ll} V & c>0 \\ 0 & c=0 \end{array}\right. } $$
Snowdrift (S) (Archetti and Scheuring 2012)	$V-\dfrac{K}{c}$	$${\left\{ \begin{array}{ll} V & c>0 \\ 0 & c=0 \end{array}\right. } $$
Hawk–Dove (HD) (Broom and Rychtář 2012)	$${\left\{ \begin{array}{ll} \frac{V}{c} & d=0 \\ 0 & d>0 \end{array}\right. } $$	$\dfrac{V-(d-1)K}{d}$

Social dilemmas are referred to in the text by the acronyms introduced in this table

We follow an approach grounded on evolutionary graph theory (Lieberman et al. 2005). The population has a corresponding evolutionary graph represented by the adjacency matrix $\textbf{W}=(w_{ij})$, with $w_{ij}$ denoting the replacement weights which determine the likelihood of individual $I_i$ replacing $I_j$ in an evolutionary step. Replacement weights are determined by the fraction of time two individuals spend together, where time is equally shared among all group members, while time spent alone contributes to an individual’s self-replacement weight:

3 $$\begin{aligned} w_{ij} = {\left\{ \begin{array}{ll} \sum \limits _{m} \sum \limits _{\mathcal {G}: i,j\in \mathcal {G}} \frac{\chi (m,\mathcal {G})}{|\mathcal {G}|-1}, & i\ne j, \\ \sum \limits _{m}\chi (m,\{i\}), & i = j. \end{array}\right. } \end{aligned}$$

Let us consider that the population goes through an evolutionary process operating on the strategies C and D used by each individual, modelled in discrete evolutionary steps. We focus on evolutionary processes with a fixed population size, corresponding to a demographic equilibrium in which births and deaths balance on average. Under this common assumption in evolutionary game theory and population dynamics, evolutionary change occurs through the replacement of strategies between individuals rather than through changes in population size. The probability that, at a given step, the strategy of an individual $I_i$ replaces that of $I_j$ is denoted by $\tau _{ij}$, which depends on the fitness of individuals and on the replacement weights. We recall the six evolutionary dynamics outlined by Pattni et al. (2017). They are categorized as birth–death (BD) when an individual is first selected for reproduction and another for removal; death–birth (DB) when this sequence is reversed; and link (L) when an edge of the evolutionary graph is selected directly. In each case, selection may operate either during the birth (B) or the death (D) step. Accordingly, the dynamics are denoted by combining these two labels. Their respective replacement probabilities $\tau _{ij}$ are summarised in Table 2. See (Pattni et al. 2017) for more detailed information on these. Differences between dynamics reflect whether reproduction events drive the process (offspring actively displace existing individuals) or whether death events create opportunities that are then filled by reproduction. The distinction between selection acting on birth or death reflects whether fitness primarily affects reproductive success or survival. Different combinations therefore capture different biological assumptions about the relative importance of reproduction and mortality in evolutionary competition.

**Table 2:** Definition of birth probabilities ($b_{i(j)}$) and death probabilities ($d_{(i)j}$), or of final replacement probability ($\tau _{ij}$), for the six distinct evolutionary dynamics described in the text

Evolutionary dynamics and replacement probabilities			
BDB	$b_i=\dfrac{F_i}{\sum _nF_n}$, $d_{ij}=\dfrac{w_{ij}}{\sum _n w_{in}}$	DBD	$d_j=\dfrac{F_j^{-1}}{\sum _nF_n^{-1}}$, $b_{ij}=\dfrac{w_{ij}}{\sum _nw_{nj}}$
DBB	$d_j=1/N$, $b_{ij}=\dfrac{w_{ij}F_i}{\sum _nw_{nj}F_n}$	BDD	$b_i=1/N$, $d_{ij}=\dfrac{w_{ij}F_j^{-1}}{\sum _nw_{in}F_n^{-1}}$
LB	$\tau _{ij}=\dfrac{w_{ij}F_i}{\sum _{n,k}w_{nk}F_n}$	LD	$\tau _{ij}=\dfrac{w_{ij}F_j^{-1}}{\sum _{n,k}w_{nk}F_k^{-1}}$

The indices denote the individuals $I_i$ giving birth and $I_j$ dying. In instances where the replacement probability is not explicitly stated, it can be derived by multiplying the respective birth and death probabilities

Finally, the fixation probability of a single cooperator in a population of defectors is denoted by $\rho ^C$, and that of a single defector in a population of cooperators by $\rho ^D$. Fixation probabilities can be calculated under fully independent movement models resorting to the proceeding explained by Broom et al. (2015) and Pattni et al. (2017). For the most part of this work, we will focus on limiting cases where fixation probabilities hold closed-form expressions. We may have four different evolutionary scenarios, depending on how the fixation probabilities of cooperators and defectors relate to the neutral fixation probability which is equal to $\rho ^{neutral}=1/N=1/(MQ)$:

- Selection favours the evolution of cooperation if $\rho ^C>\rho ^{neutral}>\rho ^D$;
- Selection favours the evolution of defection if $\rho ^D>\rho ^{neutral}>\rho ^C$;
- Selection leads to instability when both $\rho ^C>\rho ^{neutral}$ and $\rho ^D>\rho ^{neutral}$;
- Selection leads to bi-stability when both $\rho ^C<\rho ^{neutral}$ and $\rho ^D<\rho ^{neutral}$.

## Completely mixed populations

We recall the generalisation of the well-mixed population for multiplayer interactions stating that all individuals are indistinguishable and that therefore, in a group of a given size, all possible group compositions have the same probability of occurring (Broom and Rychtář 2012). This additionally means that all individuals have the same replacement weights apart perhaps from the self-replacement weights, as stated by Schimit et al. (2019).

However, this broad definition can be fulfilled for an arbitrary group size distribution. Under multiplayer interactions on networks this may come from different movement model assumptions, either because individuals have the same probability distribution over the places of the network or due to an existing high symmetry between their distributions (Broom and Rychtář 2012). For illustration, this occurs under the territorial raider model for arbitrary home fidelity values, when considering an arbitrary network where the whole population is based in the same home node, and under a complete network with each node being the home to exactly one individual. Alternative independent movement models, i.e. models with independent probability distributions for the positions, can be easily conceptualised that similarly lead to well-mixed populations, and general group size distributions can also be introduced ad hoc (Broom et al. 2019) as long as group compositions remain random.

A stricter concept of a completely mixed population is introduced by Broom and Rychtář (2012). A population following an independent movement model is said to be completely mixed, if not only individuals but also all places in the network are indistinguishable. This means that all individuals have the same uniform probability distribution over the places of the network. This will lead to a unique population with a particular group size distribution.

**Fig. 1:**
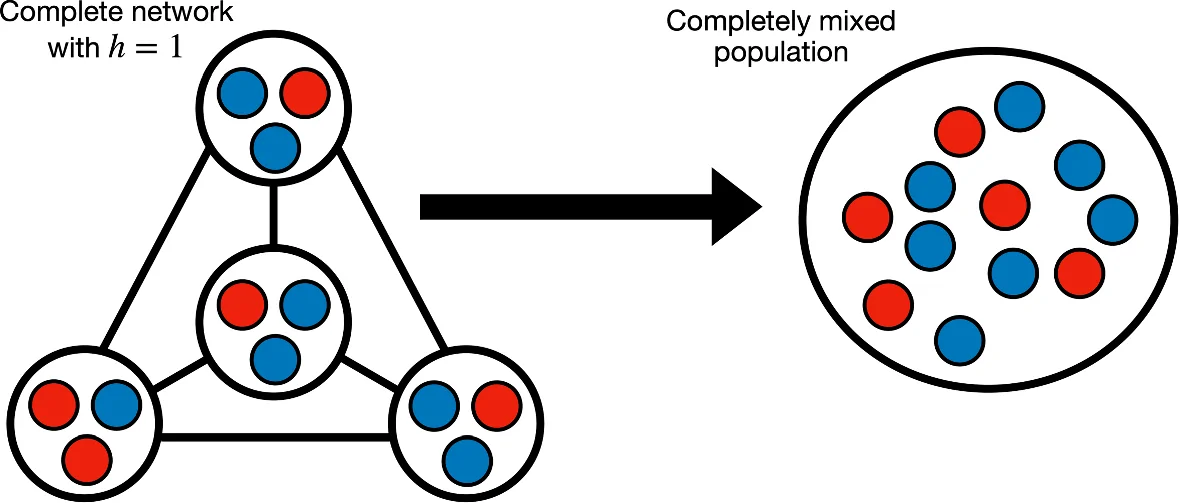
Equivalence between territorial raider model on complete network with h=1 and completely mixed population. On the left, the territorial raider model in a complete network with M=4 places and density of Q=3. Under h=1, this model is equivalent to a completely mixed population, i.e. a well-mixed population where individuals assemble in random groups of size following a binomial distribution with parameters N=M×Q=12 and p=1/M=1/4

Consider the territorial raider model introduced in the previous section. In these sections, we will focus on the emerging dynamics when home fidelity is h=1 under the territorial raider model in complete networks. In this context, all individuals have the same probability $p_{nm}=p \equiv 1/M$ of being in any place in the network. Each individual spends as much time in their home node as in any of the remaining nodes of the networks. As per its definition, this will be a completely mixed population, i.e. one where all individuals in the population are indistinguishable, and have the same probability of being in any of the (likewise indistinguishable) places of the underlying network. This makes the transition probabilities under the evolutionary dynamics the same, as well as the resulting fixation probabilities. Although the properties of this population are further elaborated throughout the paper, we now represent this equivalence schematically in Fig. 1. In this context, the fitness and replacement weights will be the same regardless of whether we have individuals split equally between communities with the same size, or one community with *N* individuals and the remaining ones empty. We will thus treat *Q* more generally as the *density* of individuals over the connected places of the complete network Q=N/M.

### Group size distribution, replacement weights and temperature

In this context, the size of the groups formed in any of the nodes of the network follows a binomial distribution with parameters *N* and 1/*M*, i.e. $B\left( N,1/M\right)$. This way, if at a given moment, a node of the network is chosen at random, the observed group has a mean size of N/M=Q. However, from the point of view of each individual, the number of other individuals in their current group will follow a binomial distribution with parameters N-1 and 1/*M*, i.e. $B\left( N-1,1/M\right)$. This way, the experienced group size of individuals will have a mean of 1+(N-1)/M=Q+1-1/M.

We call $w_1$ the replacement weights between the different individuals in a well-mixed population, and $w_0$ the self-replacement weights. In particular, in a completely mixed population, the self-replacement weights correspond to the probability of being alone:

4 $$\begin{aligned} w_0 = \left( 1- \dfrac{1}{M}\right) ^{N-1}, \end{aligned}$$

which in the limit of large populations and networks with asymptotic density $Q \equiv \lim _{N\rightarrow \infty }\lim _{M\rightarrow \infty }(N/M)$ becomes:

5 $$\begin{aligned} w_0 \rightarrow \exp (-Q). \end{aligned}$$

Therefore replacement weights between different individuals simply correspond to the probability of not being alone divided by the remaining number of individuals:

6 $$\begin{aligned} w_1 = \dfrac{1-w_0}{N-1} = \dfrac{1}{N-1}\left( 1- \left( 1- \dfrac{1}{M}\right) ^{N-1}\right) . \end{aligned}$$

The temperature of an individual is defined by Lieberman et al. (2005) as the sum of all replacement weights connected to their vertex of the evolutionary graph. Under the current model, this is reinterpreted by Broom et al. (2015) as excluding self-replacement weights, therefore depending on the graph structure and the parameter *h*. In completely mixed populations, this is equal to the following:

7 $$\begin{aligned} T = 1 - w_0 = 1- \left( 1- \dfrac{1}{M}\right) ^{N-1}. \end{aligned}$$

### Fitness under completely mixed populations

In completely mixed populations, the fitness of cooperators and defectors can be represented as $$F^C_{N_c,N_d}$$ and $$F^D_{N_c,N_d}$$, since these are determined by the strategy of the focal individual and the number of cooperators $$N_c$$ and defectors $$N_d$$ in the population. This dependence resides in the average payoff according to Eq. (2), which we represent as $$\pi ^C_{N_c,N_d}$$ and $$\pi ^D_{N_c,N_d}$$ for completely mixed populations. As previously mentioned, the number of other cooperators $c^{\prime }$ and defectors $d^{\prime }$ in the focal individual’s group follow two binomial distributions. This way, the average payoffs can simply be calculated in the following way:

8 $$\begin{aligned} \pi ^C_{N_c,N-N_c}= &  \sum _{c'=0}^{N_c-1} \left( {\begin{array}{c}N_c-1\\ c'\end{array}}\right) p^{c'}(1-p)^{N_c-1-c'}\nonumber \\ &  \sum _{d'=0}^{N-N_c} \left( {\begin{array}{c}N-N_c\\ d'\end{array}}\right) p^{d'} (1-p)^{N-N_c-d'} R^C_{c'+1,d'},\end{aligned}$$

9 $$\begin{aligned} \pi ^D_{N-N_d,N_d}= &  \sum _{c'=0}^{N-N_d} \left( {\begin{array}{c}N-N_d\\ c'\end{array}}\right) p^{c'}(1-p)^{N-N_d-c'}\nonumber \\ &  \sum _{d'=0}^{N_d-1} \left( {\begin{array}{c}N_d-1\\ d'\end{array}}\right) p^{d'} (1-p)^{N_d-1-d'} R^D_{c',d'+1}. \end{aligned}$$

We have used here each individual’s probability of being in any of the places of the network p=1/M, as introduced before.

### Fixation probabilities under well-mixed and completely mixed populations

In a well-mixed population, individuals of the same type are indistinguishable and thus hold equal replacement weights and fitness values. Consequently, the state of the population can be fully described by the number of individuals using each strategy, since all states with the same composition yield equal transition probabilities. The fixation process of a single mutant within a resident population can thus be represented as a biased random walk. This allows us to employ the closed-form expression derived by Karlin and Taylor (1975) to compute the fixation probability of one mutant cooperator in a population of defectors, and the reverse, as respectively the following:

10 $$\begin{aligned} \rho ^C=\dfrac{1}{1+ \sum _{j=1}^{N-1} \prod _{N_c=1}^j \dfrac{F^D_{N_c,N-N_c}}{F^C_{N_c,N-N_c}}}, \end{aligned}$$

11 $$\begin{aligned} \rho ^D=\dfrac{1}{1+ \sum _{j=1}^{N-1} \prod _{N_d=1}^j \dfrac{F^C_{N-N_d,N_d}}{F^D_{N-N_d,N_d}}}. \end{aligned}$$

We note that these probabilities are identical under the BDB, DBD, LB and LD dynamics because the transition probability ratios that characterise the process are identical at any given state of the population.

The DBB and BDD dynamics lead to different quantitative results as transition probability ratios in the resulting Markov chain are different from the previous four dynamics. Similarly to before, we use the closed-form expression derived by Karlin and Taylor (1975), now with the transition probabilities under the remaining dynamics DBB and BDD (obtained from Table 2). This way, we obtain the following expressions for the fixation probabilities of cooperators and defectors:

12 $$\begin{aligned} \rho ^C_{DBB/BDD}=\dfrac{1}{1{+} \sum _{j=1}^{N-1} \prod _{N_c=1}^j \dfrac{F^D_{N_c,N{-}N_c}}{F^C_{N_c,N{-}N_c}}\cdot \left( 1 {+} \dfrac{(w_0{-}w_1)\left( F^D_{N_c,N{-}N_c}{-}F^C_{N_c,N{-}N_c}\right) }{w_1T_{DBB/BDD}(N_c,N{-}N_c){+}(w_0{-}w_1)F^C_{N_c,N{-}N_c}} \right) }, \end{aligned}$$

13 $$\begin{aligned} \rho ^D_{DBB/BDD}=\dfrac{1}{1+ \sum _{j=1}^{N-1} \prod _{N_d=1}^j \dfrac{F^C_{N-N_d,N_d}}{F^D_{N-N_d,N_d}}\cdot \left( 1 {+} \dfrac{(w_0{-}w_1)\left( F^C_{N{-}N_d,N_d}{-}F^D_{N-N_d,N_d}\right) }{w_1T_{DBB/BDD}(N{-}N_d,N_d){+}(w_0{-}w_1)F^D_{N-N_d,N_d}} \right) }, \end{aligned}$$

with $T_{DBB/BDD}$ denoting positive terms as evident from their definition:

14 $$\begin{aligned} T_{DBB}(N_c,N_d) = N_c\cdot F^C_{N_c,N_d} + N_d\cdot F^D_{N_c,N_d}, \end{aligned}$$

15 $$\begin{aligned} T_{BDD}(N_c,N_d) = N_d\cdot F^C_{N_c,N_d} + N_c\cdot F^D_{N_c,N_d}. \end{aligned}$$

The DBB and BDD dynamics introduce correction coefficients into the fixation probability expressions, as it can be seen by comparing Eqs. (10), (11), (12) and (13). These correction terms come from the fact that both structure and selection act in the second event (birth for DBB and death for BDD), thus entangling weights and fitness in the replacement probabilities, contrary to what happens under the remaining dynamics. Therefore, in the DBB and BDD dynamics, the individual in the first event is chosen randomly, and in the second event their fitness is weighted by the self-replacement weight $w_0$, while the fitness of others is weighted by $w_1$. The correction coefficients in the above equations have positive denominator, and therefore, amplify differences between the transition probabilities in each ratio if $w_0>w_1$, or suppresses them if $w_1>w_0$.

In the particular case of a completely mixed population, the condition $w_0>w_1$ is met if:

16 $$\begin{aligned} \left( 1-\dfrac{1}{M}\right) ^{N-1}>\dfrac{1}{N}, \end{aligned}$$

which under large populations and networks simply corresponds to:

17 $$\begin{aligned} N>\exp (Q). \end{aligned}$$

This means that for any given finite asymptotic density $Q=\lim _{N\rightarrow \infty }\lim _{M\rightarrow \infty } (N/M)$, there is always a population size above which $w_0>w_1$. Lower densities lead more easily to $w_0>w_1$, and higher densities, typically require exponentially larger populations to do so. In those cases, individuals spend more time with themselves than with any other particular individual thus leading to the amplification of differences between transition probabilities in completely mixed populations.

In another example, namely well-mixed populations where individuals have pairwise interactions, the DBB and BDD dynamics lead to the same fixation probabilities as the other dynamics if self-replacements occur at the same rate as other replacements under neutral selection, i.e. $w_0=w_1$. If self-replacements are not possible, i.e. $w_0=0$, then the DBB and BDD dynamics suppress the differences between transition probabilities.

Finally, the limit of large home fidelity was considered in the territorial raider model for communities of the same size (Pires and Broom 2024). In that limit, even though the overall population is deeply structured, its communities are asymptotically isolated, and become well-mixed, interacting repeatedly within the same group of size *Q*. In that case, individuals are never alone, thus leading to $w_0=0$ and $w_1=1/(Q-1)$. That is the reason why it is reported that selection is suppressed in within-community events under the DBB and BDD dynamics under $h\rightarrow \infty$, a key point in explaining why cooperation evolves under all dilemmas in those dynamics, but not in the remaining dynamics.

### Fixation probabilities under weak selection

We further considered the expansion of the fixation probability equations under the limit of weak selection. Parallel expansions of fixations probabilities have been previously obtained for other systems under the weak selection limit. This includes simple pairwise games (Nowak et al. 2004) and the resulting derivation of the one-third rule (Ohtsuki et al. 2007; Lessard and Ladret 2007), but also its extension to multiplayer games under constant group size (Kurokawa and Ihara 2009; Gokhale and Traulsen 2010) and other limits of the territorial raider model (Pires and Broom 2024). Furthermore, the relation between the weak selection limit and the population size can influence the resulting fixation probability expansions and the conditions for the evolution of cooperation (Allen et al. 2021), while generating or erasing surprising finite population effects (Pires and Broom 2022). In this work, we specify that $w\cdot N\rightarrow 0$, leading to fixation probabilities in the following form:

18 $$\begin{aligned} &  \rho ^C\approx \dfrac{1}{N} + \left. \dfrac{ \partial \rho ^C}{ \partial w}\right| _{w\rightarrow 0}\cdot w, \end{aligned}$$

19 $$\begin{aligned} &  \rho ^D\approx \dfrac{1}{N} + \left. \dfrac{ \partial \rho ^D}{ \partial w}\right| _{w\rightarrow 0}\cdot w, \end{aligned}$$

where the zeroth-order term is the fixation probability under neutral selection, which can also be denoted as $\rho ^{neutral}=1/N=1/(M Q)$.

The first-order expansion terms are obtained by differentiating the fitness with respect to *w* and then setting w=0. After simplifying the resulting expressions, we obtain the following:

20 $$\begin{aligned} \left. \dfrac{ \partial \rho ^C}{ \partial w}\right| _{w\rightarrow 0}=\dfrac{1}{N^2}\sum _{N_c=1}^{N-1}(N-N_c) \left[ \pi ^C_{N_c,N-N_c} - \pi ^D_{N_c,N-N_c}\right] , \end{aligned}$$

21 $$\begin{aligned} \begin{aligned} \left. \dfrac{ \partial \rho ^D}{ \partial w}\right| _{w\rightarrow 0}&=\dfrac{1}{N^2}\sum _{N_d=1}^{N-1}(N-N_d) \left[ \pi ^D_{N-N_d,N_d}-\pi ^C_{N-N_d,N_d}\right] =\\&=\dfrac{1}{N^2}\sum _{N_c=1}^{N-1}N_c \left[ \pi ^D_{N_c,N-N_c} - \pi ^C_{N_c,N-N_c} \right] . \end{aligned} \end{aligned}$$

Performing the same expansion for the DBB and BDD dynamics, we observe that the first-order term would simply have a correction coefficient added to it:

22 $$\begin{aligned} \left. \dfrac{ \partial \rho ^*_{DBB/BDD}}{ \partial w}\right| _{w\rightarrow 0} = \left( 1 + \dfrac{w_0-w_1}{w_1N + (w_0-w_1)} \right) \cdot \left. \dfrac{ \partial \rho ^*}{ \partial w}\right| _{w\rightarrow 0}, \end{aligned}$$

where we have used the general strategy index ∗ to represent the fixation probability of any of the existing strategies. Under large completely mixed populations, this simply becomes 1+exp(-Q).

The correction coefficient only impacts the evolutionary dynamics by amplifying/suppressing fixation in the already reported way. It does not affect the sign of the first-order term of the expansion, and thus the DBB and BDD dynamics have no impact on the conditions for cooperation to evolve explored in the following section.

## Evolution of cooperation in completely mixed populations

### General social dilemmas in completely mixed populations

We consider a selection of social dilemmas identified by Broom et al. (2019). These multiplayer games capture various scenarios involving the production of public goods with distinct characteristics, or the consumption of pre-existing commons. Using Eqs. (8) and (9), derived for completely mixed populations, we analyse the average payoff of cooperators and defectors in five social dilemmas and present the corresponding results in Table 3. The detailed derivations are provided in section 1 of the supplementary information.

**Table 3:** Exact average payoff obtained under multiplayer social dilemmas in completely mixed populations

Game	$\pi ^C_{N_c,N-N_c}$	$\pi ^D_{N_c,N-N_c}$
CPD	$\dfrac{N_c-1}{N-1} \left[ 1-\left( 1-\dfrac{1}{M}\right) ^{N-1}\right] V-K$	$\dfrac{N_c}{N-1}\left[ 1-\left( 1-\dfrac{1}{M}\right) ^{N-1}\right] V$
PD	$\dfrac{M}{N} \left[ 1-\left( 1-\dfrac{1}{M}\right) ^{N}\right] V +\dfrac{N_c-1}{N-1}\left[ 1-\dfrac{M}{N}\left( 1- \left( 1-\dfrac{1}{M}\right) ^{N}\right) \right] V -K$	$\dfrac{N_c}{N-1} \left[ 1-\dfrac{M}{N}\left( 1- \left( 1-\dfrac{1}{M}\right) ^{N}\right) \right] V$
VD	V-K	$\left[ 1- \left( 1-\dfrac{1}{M}\right) ^{N_c}\right] V$
S	$V - \dfrac{M}{ N_c}\left[ 1- \left( 1-\dfrac{1}{M}\right) ^{N_c} \right] K$	$\left[ 1- \left( 1-\dfrac{1}{M}\right) ^{N_c}\right] V$
HD	$\dfrac{M}{N_c} \left( 1-\dfrac{1}{M}\right) ^{N-N_c}\cdot \left[ 1-\left( 1-\dfrac{1}{M}\right) ^{N_c}\right] V$	$\dfrac{M}{N-N_c}\left[ 1-\left( 1-\dfrac{1}{M}\right) ^{N-N_c}\right] (V+K)-K$

The fitness of both cooperators and defectors under the CPD and the PD depends linearly on the frequency of each strategy in the population. This means that both multiplayer games in completely mixed populations have equivalent pairwise games in well-mixed populations. In the CPD, the fitness of defectors is larger than that of cooperators for any positive value of *V* and *K*. In the PD, defectors have larger fitness if *V*/*K* is larger than a critical value. Even though both games are motivated as extensions of the pairwise Prisoner’s Dilemma to multiplayer interactions, they hold significantly different results. This comes directly from the fact that cooperators benefit from their own contributions in the PD, contrary to what happens in the CPD.

The VD, S and HD hold different results, as the strategy with larger fitness depends not only on the payoff parameters *V* and *K*, but also on the state, i.e. composition, of the population, determined from the number of cooperators $N_c$. We compare the payoffs received by cooperators and defectors and summarise the results in Table 4, considering V>0 and K>0 and the approximation of large *N*. We identify three distinct scenarios: either one strategy yields a higher average payoff than the other regardless of the state of the population and is thus considered advantaged, or the relative performance of the two strategies depends on the state of the population.

**Table 4:** Comparison of average payoff received by cooperators and defectors under multiplayer social dilemmas in completely mixed populations

Game	Defectors advantaged	State-dependent	Cooperators advantaged
CPD	Always	-	-
PD	$V/K<\dfrac{1}{1-\exp {(-Q)}}$	-	$V/K>\dfrac{1}{1-\exp {(-Q)}}$
VD	V/K<1	1<V/K<exp(Q)	V/K>exp(Q)
S	V/K<1	$1<V/K<\dfrac{\exp {(Q)}-1}{Q}$	$V/K>\dfrac{\exp {(Q)}-1}{Q}$
HD	$V/K>\dfrac{Q-1+\exp (-Q)}{1-(Q+1)\exp (-Q)}$	$V/K<\dfrac{Q-1+\exp (-Q)}{1-(Q+1)\exp (-Q)}$	-

In public goods dilemmas, defectors are advantaged under low enough rewards, whereas high enough rewards lead to the advantage of cooperators (except in the CPD). Under the VD and the S there is an additional region for intermediate values of the reward-to-cost ratio where the strategy with higher payoff depends on the composition of the population. Under the HD, cooperators are never advantageous irrespective of the population state. Instead, we observe state-dependent advantages under low reward values, and defectors becoming advantageous for large rewards. In all observed state-dependent regions, cooperators do better amongst defectors and defectors do better amongst cooperators, potentially leading to mutual fixation and the instability of both strategies.

The CPD and PD are games where one of the strategies is always advantageous irrespective of population composition. Therefore the evolutionary dynamics considered don’t change the evolutionary outcomes and the advantageous strategy will always fixate with probability higher than the neutral one. However, under the VD, the S and the HD, we observe values of payoff parameters leading to state-dependent advantages. In those cases, it is not trivial which of the strategies fixates on the other, as it is stated by Taylor et al. (2004) for pairwise games in well-mixed populations. In these cases, choosing the DBB or BDD dynamics might also move the boundaries under which each strategy fixates successfully. To further study these boundaries, we take into consideration the weak selection limit.

### Rules of cooperation under large completely mixed populations

We apply the average payoff functions obtained for five social dilemmas in Sect. 4.1 onto the fixation probability equations presented in Sect. 3.4, where we have considered the weak selection limit $w\cdot N \rightarrow 0$ in order to obtain exact conditions for the different evolutionary outcomes. The limits of large populations and large networks were also considered to simplify those expressions, and we denote the asymptotic density of individuals as $Q=\lim _{N\rightarrow \infty }\lim _{M\rightarrow \infty }(N/M)$. The conditions are summarised in Table 5, as well as the evolutionary scenarios they may lead to. We recall that selection favours the evolution of cooperation if $\rho ^C>\rho ^{neutral}>\rho ^D$, it favours the evolution of defection if $\rho ^D>\rho ^{neutral}>\rho ^C$ and leads to instability when both $\rho ^C>\rho ^{neutral}$ and $\rho ^D>\rho ^{neutral}$. Selection leading to bi-stability was not observed in any of the five social dilemmas in completely mixed populations. The derivation of these conditions is done in section 2 of the supplementary information.

**Table 5:** Conditions for the evolution of multiplayer cooperation under large completely mixed populations with asymptotic density *Q*

Game	$\rho ^C>\rho ^{neutral}$	$\rho ^D>\rho ^{neutral}$	Selection favours
CPD	Never	Always	Defection
PD	$V/K>\dfrac{Q}{1-\exp {(-Q)}}$	$V/K<\dfrac{Q}{1-\exp {(-Q)}}$	Defection, Cooperation
VD	$V/K>\dfrac{Q/2}{1-\dfrac{1-\exp {(-Q)}}{Q}}$	$V/K<\dfrac{Q^2/2}{1-\exp {(-Q)}(Q+1)}$	Defection, Instability, Cooperation
S	$V/K>\dfrac{L_1(Q)-1+\dfrac{1-\exp {(-Q)}}{Q}}{1-\dfrac{1-\exp {(-Q)}}{Q}}$	$V/K<\dfrac{Q-1+\exp {(-Q)}}{1-\exp {(-Q)}(Q+1)}$	Defection, Instability, Cooperation
HD	$V/K<\dfrac{\dfrac{Q}{2}-1+\dfrac{1-\exp {(-Q)}}{Q}}{1-\exp {(-Q)}\cdot (L_2(Q)+1)}$	$V/K>\dfrac{\dfrac{Q}{2}+1-L_1(Q)-\dfrac{1-\exp {(-Q)}}{Q}}{L_{1}(Q)-(1-\exp {(-Q)})}$	Cooperation, Instability, Defection

In some of these conditions, the functions $L_1(Q)$ and $L_2(Q)$ were used, which are defined based on the limit of the difference between the harmonic series, i.e. $H_{N-1}=\sum _{i=1}^{N-1}1/i$, and two other geometric-harmonic series:

23 $$\begin{aligned} L_{1}(Q)=\lim _{M\rightarrow \infty } \left[ H_{M\times Q -1} -\sum _{i=1}^{M\times Q-1}\dfrac{\left( 1-\dfrac{1}{M} \right) ^i}{i}\right] , \end{aligned}$$

24 $$\begin{aligned} L_{2}(Q)=\lim _{M\rightarrow \infty } \left[ \sum _{i=1}^{M\times Q-1}\dfrac{\left( 1-\dfrac{1}{M} \right) ^{-i}}{i} - H_{M\times Q -1} \right] . \end{aligned}$$

**Fig. 2:**
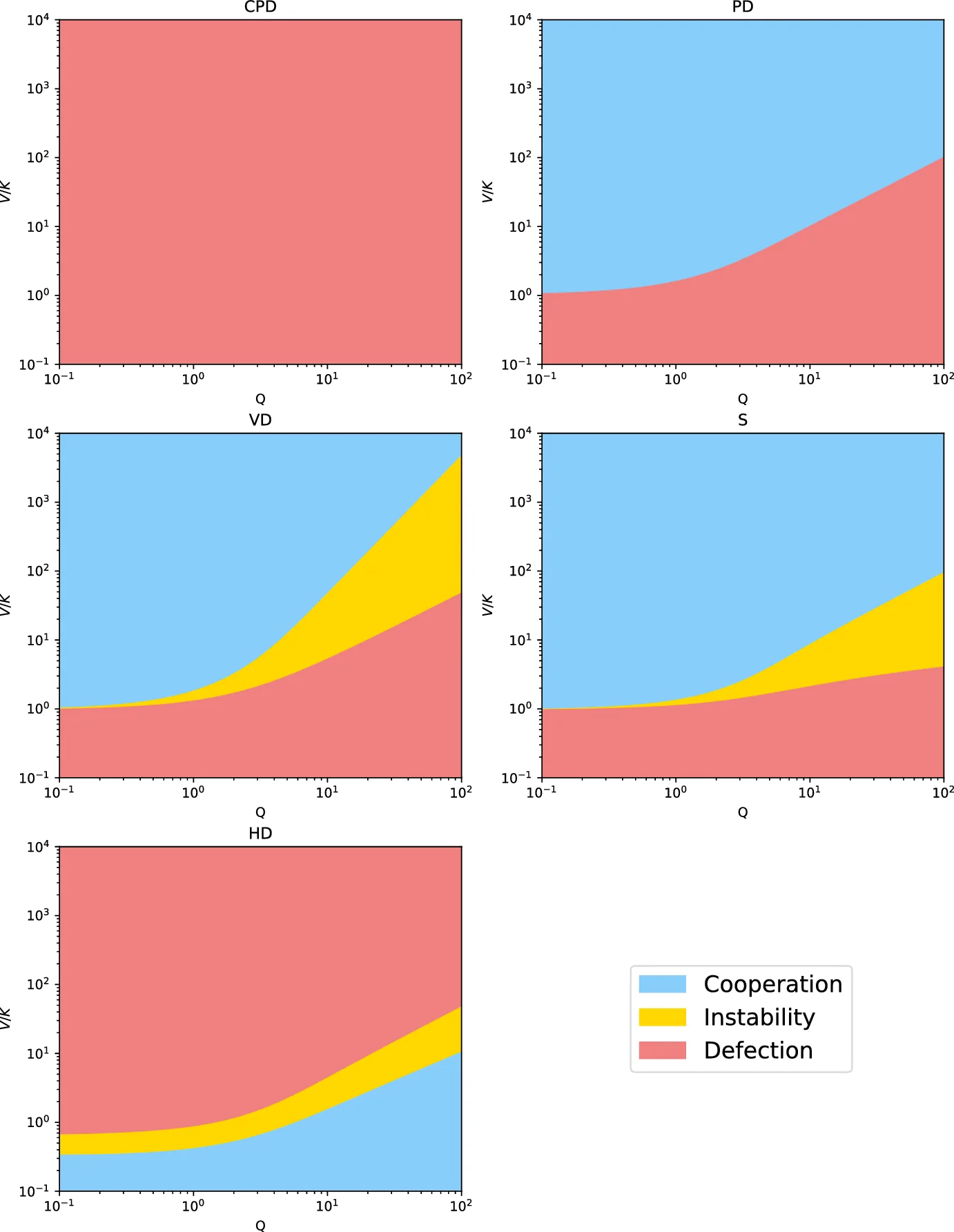
Evolutionary outcomes obtained for different values of the reward-to-cost ratio *V*/*K* in large completely mixed populations with asymptotic density *Q*. The figure was built based on the critical values from the conditions in Table 5

These two functions are both zero for asymptotically low density *Q*, i.e. $L_1(0)=L_2(0)=0$, and both increase with *Q*, thus being positive functions. $L_1(Q)$ increases sublinearly with *Q*, and takes, for example, values $L_1(1)\approx 0.80$, $L_1(2)\approx 1.32$, $L_1(3)\approx 1.69$, and $L_1(20)\approx 3.57$. $L_2(Q)$ increases supralinearly with *Q*, and takes, for example, $L_2(1)\approx 1.32$, $L_2(2)\approx 3.68$, $L_2(3)\approx 8.26$, and $L_2(20)\approx 2.56\times 10^7$. However, the result of $\exp {(-Q)}\cdot (L_2(Q)+1)$ is 1 for Q=0 and decreases monotonically to zero as *Q* increases. The geometric-harmonic series used here also comes up when the HD game is explored by Haq et al. (2024) in complete territorial networks with arbitrary *h* for the particular case where each node hosts one individual (Q=1).

The rules for the evolution of cooperation presented in Table 5 can be intricate to interpret, so we represent them visually in Fig. 2 as regions where selection favours cooperation, instability, or defection for different values of the density *Q* and the reward-to-cost ratio. We do not represent cases of bi-stability as these were not found. Under large populations and networks, the conditions depend only on the asymptotic value of the ratio Q=N/M, which we named the density of the population, corresponding to the community size under large home fidelity (Pires and Broom 2024), or the subpopulation size used in other approaches (Pattni et al. 2017).

Cooperation can never evolve under the CPD in completely mixed populations. However, in the remaining public goods dilemmas, i.e. the PD, the VD, and the S, it always has a chance of evolving if the value of the reward-to-cost ratio is larger than a critical value determined by the density of the population. In the HD, it does so if the reward-to-cost ratio is lower than a critical value. The critical values of *V*/*K* increase in all games. This assures that higher density of individuals hinders the chances of cooperation evolving under public goods dilemmas, but affects them positively under the HD.

In Fig. 2, the critical value of *V*/*K* under the Prisoner’s Dilemma starts at 1 for asymptotically low density Q→0 and increases for larger densities, approaching the value of *Q* from above. In this game, V/K>Q means that in groups of up to size *Q*, the share of the reward reaped by a cooperator is larger than the cost paid by them. Since cooperators benefit from their contributions to the public good, cooperation thus evolves only when a large share of the interactions in which individuals partake do not represent a dilemma.

In the Volunteer’s Dilemma, the critical values start at 1 as well, but a gap opens between successful fixation of cooperators and the failed fixation of defectors. Even though cooperators fixate successfully for rewards of approximately *Q*/2 for larger densities, defectors only fail to fixate for rewards above approximately $Q^2/2$. The region of instability increases its size quadratically. Defection becomes unstable more easily, but cooperation very rarely becomes stable. This is so because under high density of individuals, 1) a small number of cooperators are advantageous in populations of defectors because they always get a full reward contrary to defectors; and 2) a small number of defectors are advantageous in populations of cooperators because they often get the rewards from cooperators without paying a cost.

In the Snowdrift game, the critical values start at 1 once more, and there is a gap opened which is narrower than under the VD. For large densities, cooperators fixate successfully for rewards growing sublinearly on *Q* and defectors become unstable for rewards close to *Q*. This results are better than those obtained under all previous dilemmas. This is due cooperators always benefiting from the full reward, which increases their chances of fixating on defectors, as seen in the VD. However, under the VD they pay a lower cost if other cooperators are present in the group as well, therefore making their own populations much more resistant to invading defectors, when compared to the VD.

Finally, in the Hawk-Dove game, defectors try to monopolise the rewards by resorting to costly aggression. Therefore, if the reward-to-cost ratio is high enough, defectors hold an advantage because their aggressive behaviour pays off. The critical values of the rewards increase with *Q*, because in larger groups, it is more likely that several defectors will be present in the same group, thus leading to higher costs of fighting and lower chances of monopolising the reward, improving the chances of cooperators evolving.

## Evolution of cooperation in networks of communities

### Comparison between community-structured and completely mixed populations

The results obtained for completely mixed populations may serve as a useful benchmark for comparison with those arising in structured populations. As we have shown above, the completely mixed case emerges naturally from considering the territorial raider model in complete networks with h=1. Another limiting case of this model arises for any network as $h\rightarrow \infty$, which has been examined in detail by Pires and Broom (2024). In that work, the resulting dynamics were shown to correspond to a nested Moran process, in which fixation occurs first at the within-community level and subsequently at the population level. Moreover, conditions for the evolution of cooperation were derived there for ten distinct social dilemmas, encompassing the five multiplayer dilemmas analysed in the present paper This makes it particularly relevant to compare the outcomes obtained under community structure with those observed in completely mixed populations. Here, we restrict our comparison to integer values of density Q≤2, for which the community-structured results remain valid.

First of all, cooperation evolves under the CPD in community-structured populations when the DBB and BDD dynamics are considered, whereas it never does in completely mixed populations. This highlights that community structure can indeed be a powerful promoter of cooperation, allowing for cooperation to evolve in dilemmas which are particularly challenging and have no chance of evolving cooperation in mixed populations. In the remaining games, cooperation may evolve in completely mixed populations. However, there are gaps between the parameter regions for which cooperation evolves under completely mixed and community-structured populations.

Under the PD, only one strategy fixates successfully at a time. The critical reward-to-cost ratio *V*/*K* above which cooperators fixate successfully under completely mixed populations is larger than *Q* but approaches that value for larger densities. This can be compared to *Q* and *Q*/2 obtained under community-structured populations for different dynamics. Community structure always leads to a wider parameter region where cooperation fixates successfully. When communities become larger, the critical values obtained in the BDB, DBD, LB, and LD dynamics under the two organisations of populations converge to the same value *Q*. In those dynamics, cooperation can only evolve if cooperators have a direct individual benefit in cooperating. However, comparing the two structures under the DBB and BDD dynamics, the gap between the two critical values is considerable and maintained for large *Q* (values become *Q* and *Q*/2). This is explained by the fact that these dynamics are sensitive to the viscosity of evolutionary processes on structured populations, as originally noted (Ohtsuki et al. 2006) and further analysed for this multiplayer game framework (Pires and Broom 2024).

In the remaining games VD, S, and HD, it’s possible to observe cooperators and defectors fixating successfully on populations of the other strategy for the same parameter values – see instability regions in Fig. 2. In comparison, under weak selection and large networks of community-structured populations, there is only one single strategy fixating successfully on the other for each choice of parameters.

In particular under the VD, community-structured populations allow for the evolution of cooperation if V/K>Q for the BDB and equivalent dynamics. This value is therefore in the instability region of completely mixed populations, between *Q*/2 and $Q^2/2$ under large densities. Under these dynamics, community structure widely stabilises cooperation when $Q<V/K<Q^2/2$, but it might instead stabilise defection when Q/2<V/K<Q. However, under the DBB and BDD dynamics, community structure guarantees the evolution and stability of cooperation if V/K>Q/2, which covers the whole region of evolution of cooperation and instability, and part of the region of defection under completely mixed populations (see for example Q=2 and V/K=1.5).

Under the Snowdrift game in community-structured populations with the BDB and equivalent dynamics, the critical value of the reward-to-cost ratio is the sum of the harmonic series with *Q* terms. Similar to the VD, this value falls on the instability region of completely mixed populations, meaning that community structure sometimes stabilises cooperation but other times defection in comparison to completely mixed populations. However, under the DBB and BDD dynamics, the critical value obtained under community-structured populations always falls on the region of evolution of defection of completely mixed populations, thus having a parallel effect to that noted under the VD.

Overall, these results obtained for public goods dilemmas follow the general trend that the DBB and BDD dynamics are much more sensitive to the effects of population structure on evolutionary processes than the remaining dynamics studied. Community structure under the BDB and equivalent dynamics always reduced the areas under which defectors fixated successfully, but sometimes at the expense of also decreasing the areas under which cooperators fixated successfully. However, community structure under DBB and BDD dynamics always extended the areas under which cooperators fixated successfully together with reducing those under which defectors did.

Under the Hawk-Dove game, the relation between critical values is not entirely consistent. Under the BDB and equivalent dynamics, community structure starts by reducing the size of the region of *V*/*K* where cooperation evolves when Q=2, and moves on to fall on the instability region for Q≥3. Under the DBB and BDD dynamics, community structure starts by assuring that cooperators evolve regardless of *V*/*K* under Q=2. For Q=3, the critical reward-to-cost ratio falls on a finite value on the defection area of completely mixed populations thus representing a positive effect to the evolution of cooperation. Finally, for Q≥4 it falls on the instability region thus sometimes guaranteeing the stability of cooperation, and others the stability of defection. This overall suggests that the effect of community structure on the evolution of cooperation under the HD is not strictly beneficial.

### Fair comparisons

Broom and Rychtář (2012) define a comparison between completely mixed and structured population models as a *fair comparison* when the mean group size is identical in both populations. This definition for evaluating evolutionary outcomes in structured populations is formulated to eliminate potential confounding effects arising from differences in experienced group size that could influence fixation probabilities, thereby isolating the impact of population structure itself.

As discussed is Sect. 3, the average group size experienced by each individual in a completely mixed population is 1+(N-1)/M, which in the limit of large populations and large networks reduces to Q+1. This implies that, in large populations, individuals experience an average group size one unit greater than the density of the population. To enable a fair comparison with the results obtained under large home fidelity, where the mean experienced group size simply equals the density $Q^{\prime }$, due to the absence variance, we instead compare these results with those from completely mixed populations of lower density $Q=Q^{\prime }-1$, which yield the same mean experienced group size $Q^{\prime }$. The two populations, of equal size $N=N^{\prime }$ are embedded in networks of different sizes *M* and $M^{\prime }$, both large but related by $$M=M'\left( 1+\frac{1}{Q}\right) $$.

This implies that the benefits obtained by cooperators under community structure, as described in the previous section, would be slightly attenuated under the PD, VD and S games, since adopting a lower density for the benchmark completely mixed population ($Q=Q^{\prime }-1$ instead of $Q=Q^{\prime }$) results in broader parameter regions favouring the evolution of cooperation. Nonetheless, even after accounting for these effects, the DBB and BDD dynamics consistently show that community structure increases the regions where cooperation evolves across all public goods dilemmas, including the CPD. The impact of community structure in the BDB and equivalent dynamics was relatively low under fair comparisons, which means that the previously observed improvements are likely associated to changes in the experienced average group size. In some cases, community structure was even detrimental to the evolution of cooperation. Specifically, in the PD for all values of *Q*, and in the VD and S games when Q=2. In the HD game, lower *Q* reduces the likelihood of cooperation evolving in the benchmark completely mixed population, thereby amplifying the relative advantage of community structure under fair comparison. Overall, these results demonstrate that the positive effects of community structure on the evolution of cooperation under DBB and BDD dynamics are robust to fair comparisons and therefore represent a solid outcome.

### Evolution of cooperation in networks of mixing communities

**Fig. 3:**
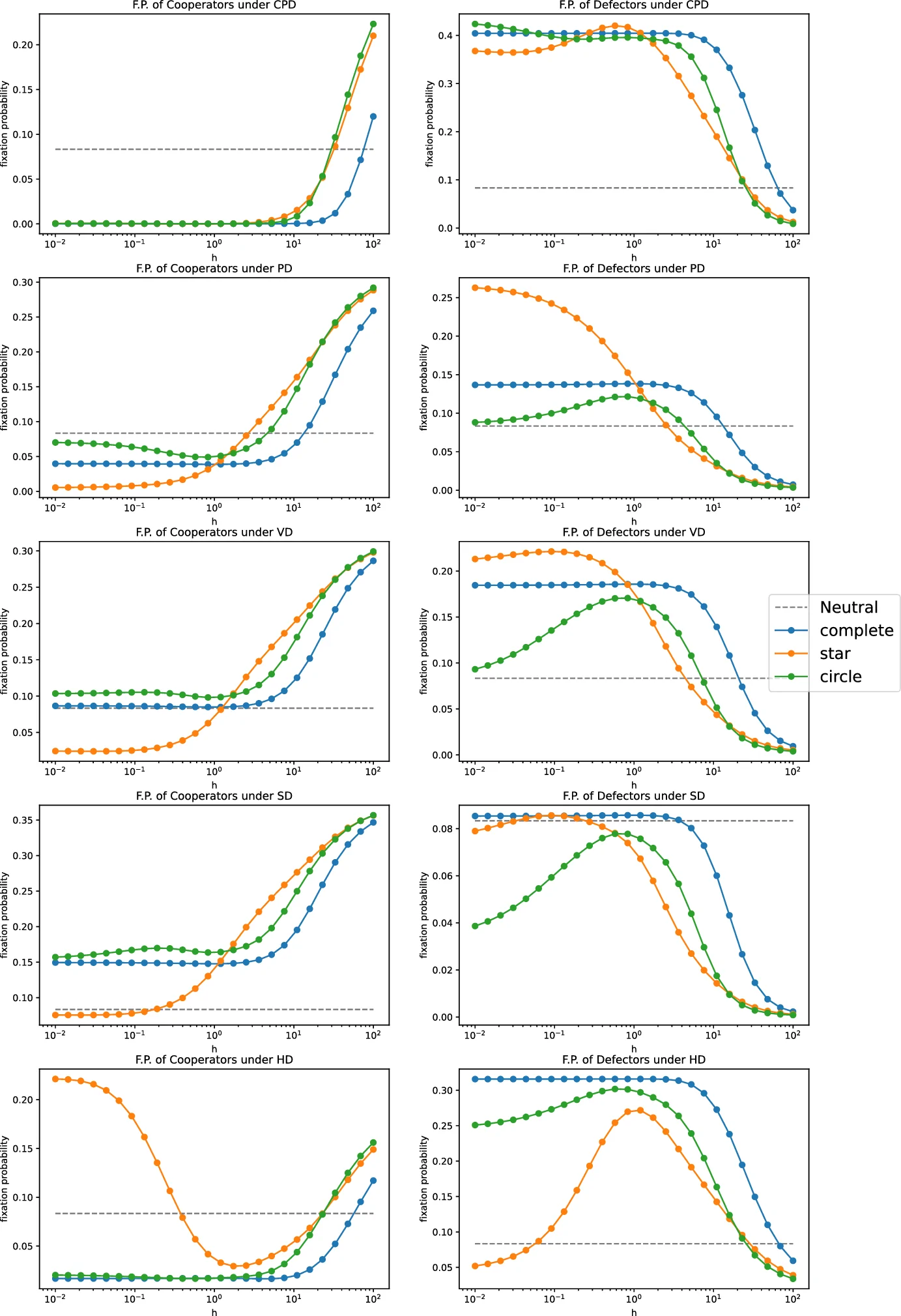
Fixation probabilities of cooperators (left) and defectors (right) under five different social dilemmas (rows) for different values of home fidelity *h*. Each coloured line represents a different topology of a network of size M=6, and communities of size Q=2. The process used was the DBB dynamics, and payoff parameters are w=0.4, V=2, and K=1. Two cases of interest are 1) completely mixed populations, i.e. complete networks under h=1, and 2) community-structured populations, i.e. all topologies under $h\rightarrow \infty$

In this section, we evaluate the results of fixation processes for wider values of home fidelity and different network topologies with the purpose of bridging the result obtained under community structure ($h\rightarrow \infty$) and in completely mixed populations (h=1 in complete networks). In Fig. 3, we observe the fixation probabilities of cooperators (left) and defectors (right) for different social dilemmas (rows).

As noted by Pires and Broom (2024), under large home fidelity all topologies converge to the same asymptotic fixation probability. We observe this in Fig. 3, even though the rate of convergence is slower under the CPD in complete networks. Overall, we observe that structure becomes relevant under lower values of *h*, therefore leading to the divergence of fixation probability curves in different topologies. Lower home fidelity hinders cooperation under public goods dilemmas. Under the CPD, it drops its fixation probability to zero under all topologies, but not under the remaining dilemmas, where sometimes cooperation succeeds for any value of *h*.

We observe that in the four public goods dilemmas, cooperators do better in star and circle networks than in complete networks for values h>1. The star and circle networks have lower average degree (2×(1-1/M) and 2 respectively), than the complete (M-1), thus leading to a rescaling of the probabilities of staying within their community. Therefore, scenarios with higher subpopulation temperature (Pattni et al. 2017), i.e. probability of interacting with individuals outside their community, might be overall detrimental to the communal cooperation effects noted by Pires and Broom (2024). This is also what happens when no subpopulations are considered (Q=1), in that case because lower temperatures translate into individuals being mainly alone and cooperators doing better (although never evolving) because defectors cannot take advantage of them (Schimit et al. 2022).

Under all public goods dilemmas apart from the CPD, cooperators under lower home fidelity do worse in star networks. This is associated with the fact that under h<1 in star networks, groups tend to assemble in the centre and thus increase the average experienced group size. Low average group size has been shown as another good determinant of cooperation under public goods dilemmas (Schimit et al. 2019, 2022), similar to what we observe here accounting for communities. This effect is particularly present in spatial networks with high node degree variance where large groups assemble in the hubs of the network (Schimit et al. 2019, 2022) and there is a general loss of the beneficial effects of highly local populations previous noted (Pires and Broom 2024).

The results obtained under the Hawk-Dove game show that low mixing of groups is beneficial to cooperators. This occurs both when $h\rightarrow \infty$ in all networks and h→0 in the star network. Moreover, it is previously reported that cooperators under the HD game benefit from being in larger communities when there is no mixing (Pires and Broom 2024), which might explain why they thrive the most under extreme low fidelity in star networks. In all networks when h≈1 there is high mixing of individuals and those benefits are lost.

**Fig. 4:**
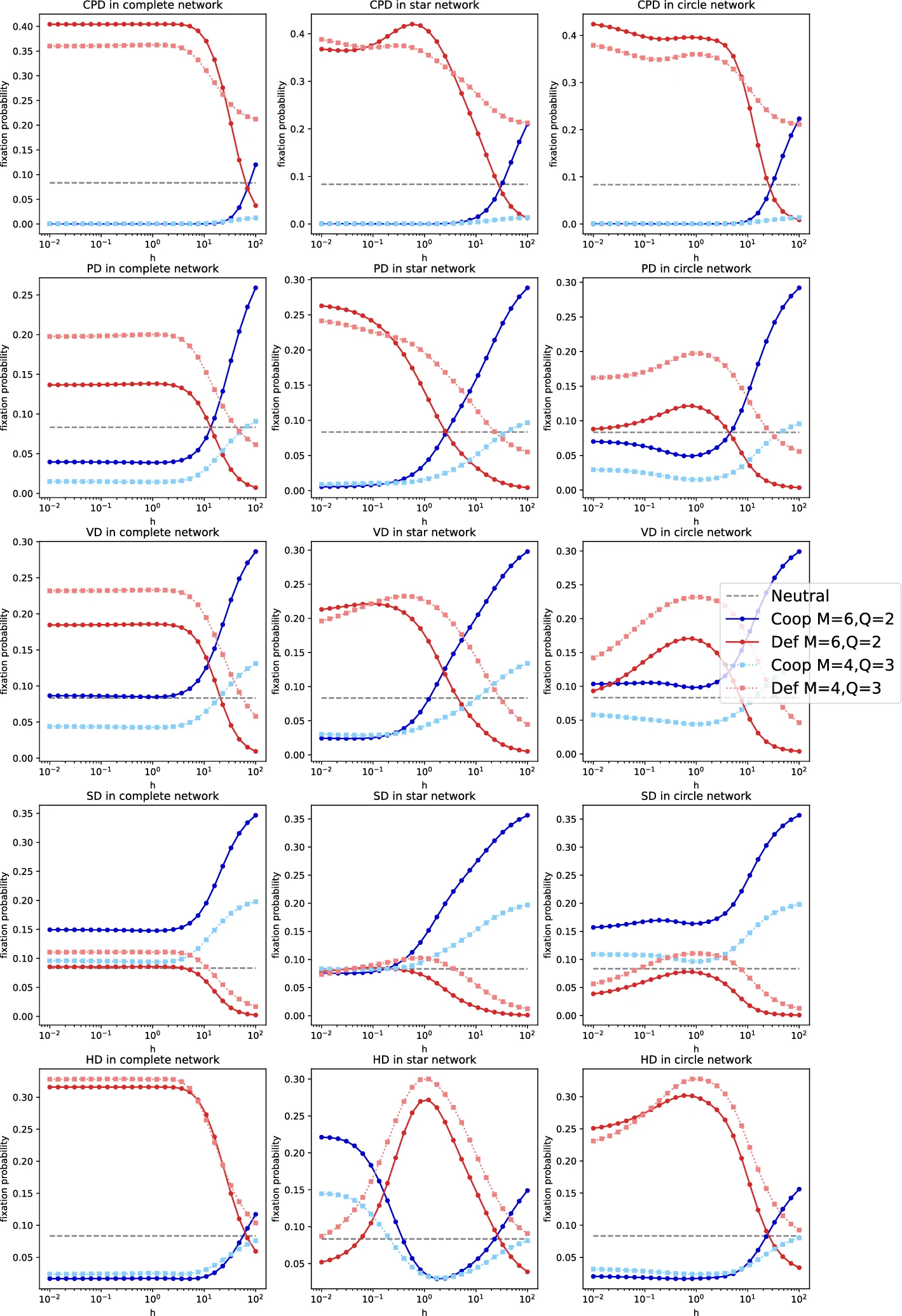
Fixation probabilities under complete (left), star (centre), and circle (right) networks for five different social dilemmas (rows). Fixation probabilities of cooperators (blue lines) and defectors (red lines) are shown in the same plots for different values of home fidelity *h* and two cases with equal population size: one with network size M=6 and community size Q=2 (circle markers), and another one with network size M=4 and community size Q=3 (triangle markers). The process used was the DBB dynamics, and payoff parameters are w=0.4, V=2, and K=1

Figure 4 shows the results obtained in different possible organisations of populations of size N=12 under this framework. This includes communities of size 2 distributed over 6 nodes and communities of size 3 distributed over 4 nodes of complete, star and circle networks.

It is clear that under public goods dilemmas, cooperators fixate successfully for wider values of *h* in larger networks (M=6) of smaller communities (Q=2). This was already noted for the limit of large home fidelity (Pires and Broom 2024). In the particular case of the CPD, cooperators never get to evolve when M=4 and Q=3 for this choice of parameters. Under large home fidelity, it is shown by Pires and Broom (2024) that cooperation evolves if the reward-to-cost ratio respects the following condition: $V/K>(Q-1)\cdot (1-\frac{2}{MQ})/(1-\frac{2(Q-1)}{MQ})$. Under Q=2 and M=6 this is equivalent to V/K>1. Under Q=3 and M=4, this is equivalent to V/K>2.5. This explains why under V=2 and K=1 we observe cooperation succeeding in one of the community-organised populations, but not on the other. Here, we further observe that the beneficial effect of organising into smaller communities is generally maintained for lower home fidelity values.

Moreover, it can be observed in Fig. 4 that both cooperators and defectors fixate on each other for the same values of the parameters under wide intervals of *h*. This is generally not observed in the CPD and the PD, but is overall present in the VD, the S, and HD games. These results agree with the previous mathematical analysis of completely mixed populations under weak selection, where such cases were possible only in these three games.

## Discussion

In this work, we have developed key concepts for understanding completely mixed populations. In such populations, all individuals are indistinguishable, as in classical well-mixed populations, and all places in the underlying spatial network are likewise indistinguishable. This makes them a stricter form of the well-mixed population, generalised to multiplayer games with variable group sizes. Completely mixed populations thus serve as an appropriate benchmark for evaluating evolutionary outcomes in structured populations with multiplayer interactions, as they represent the ultimate unstructured setting. We have used the territorial raider model as a useful independent movement model, whose case with complete network with h=1 leads to this completely mixed population and in order to facilitate comparison with previous extensive results in related studies. We have derived fundamental characteristics of these populations, including group size distribution, fitness functions and fixation probabilities, and applied these results to five distinct social dilemmas, obtaining the corresponding conditions for the evolution of cooperation. We then compared these cooperation rules with those previously derived for community-structured populations, clarifying how to perform fair comparisons between structured and unstructured populations.

We have proved that cooperation does not evolve under the Charitable Prisoner’s Dilemma (CPD) in completely mixed populations, as detailed in section 2 of the supplementary information. This is in line with what has been noted in populations playing the pairwise Prisoner’s Dilemma, where cooperation only evolves in the presence of social structure features such as low average degree (Ohtsuki et al. 2006), scale-free properties (Santos and Pacheco 2005; Santos et al. 2006b) together with high clustering (Assenza et al. 2008), high degree heterogeneity (Santos et al. 2006a), strong pair ties (Allen et al. 2017), or in the presence of alternative mechanisms (Nowak 2006). However, we have shown that cooperation evolves under some completely mixed populations in the remaining multiplayer dilemmas. The conditions for its evolution are typically characterised by a critical reward-to-cost ratio: cooperation evolves when this ratio exceeds the critical value in public goods dilemmas, and when it falls below it in the Hawk–Dove game. The critical value increases with population density, indicating that cooperation is favoured at lower densities in public goods dilemmas and at higher densities in the Hawk–Dove game. This thus shows that in order to come to a conclusion on the positive effects of spatial structure features on the evolution of multiplayer cooperation the results should be compared to the appropriate unstructured population, as this might allow for the evolution of cooperation under the chosen dilemma. We highlight that the reinterpretation of the completely mixed populations within models of multiplayer games with non-spatial structure, such as those obtained in pairwise social interaction graphs (Pacheco et al. 2009; Archetti and Scheuring 2012; Santos et al. 2008, 2006a; Souza et al. 2009) and hypergraphs (Alvarez-Rodriguez et al. 2021; Civilini et al. 2021, 2024), would allow for fair comparisons to be performed in the future.

Completely mixed populations also exhibit the simultaneous evolutionary instability of the two strategies in the Volunteer’s Dilemma, the Snowdrift game and the Hawk-Dove game. The first two games represent non-rivalrous public goods, which have shown a high propensity to sustain the fixation of cooperation in complex social interaction networks (Archetti and Scheuring 2012; Santos et al. 2008; Gómez-Gardeñes et al. 2011b). These multiplayer games are those which allow for cooperators to hold an evolutionary advantage in a population of defectors and vice-versa. This leads to two unstable strategies, similarly to what was originally observed in some pairwise games (Taylor et al. 2004). The range of parameter values for which this happened was particularly wide under the Volunteer’s Dilemma. This further highlights that results should consider not only whether cooperation fixates successfully, but simultaneously that the fixation of defection is unsuccessful.

We have compared the general rules for multiplayer cooperation obtained under community structure (Pires and Broom 2024) with those derived here for completely mixed populations. Overall, community structure promotes the evolution of cooperation across all public goods dilemmas. This effect is particularly pronounced under the DBB and BDD dynamics, which are known to be sensitive to the effects of population structure on evolution in both pairwise (Ohtsuki et al. 2006; Hauert and Imhof 2012; Allen et al. 2017) and multiplayer interactions (Pattni et al. 2015, 2017). Under these dynamics, community structure consistently expands the parameter regions in which cooperators successfully fixate and reduces those favouring defectors. In contrast, the positive impact of community structure is generally weaker under the BDB, DBD, LB and LD dynamics.

We have further performed fair comparisons between the two populations by adjusting the density of individuals in the benchmark completely mixed population to eliminate confounding effects arising from differences in average group size. Our results show that the positive impact of community structure on the evolution of cooperation under DBB and BDD dynamics remains robust under fair comparisons, representing a consistent and solid outcome. In contrast, the BDB, DBD, LB and LD dynamics produced mixed results, at times offering slight improvements and at others reducing the conditions under which cooperation evolves. We thus reinforce the conclusion that the latter dynamics are relatively insensitive to the effects of population structure on the evolution of both pairwise and multiplayer cooperation. Under alternative mechanisms, such as the co-evolution of assortative behaviour among cooperators through conditional movement (Pires et al. 2023), the specific choice of evolutionary dynamics plays only a minor role.

The evolution of cooperation under public goods dilemmas benefits from the organisation of populations into smaller, locally interacting social groups. On a practical side, this may inform policies in systems of public goods governance where public goods games have proved to be useful, such as AI monitoring (Alalawi et al. 2026), disease evolution and spread (Morison et al. 2025), environmental governance (Couto et al. 2020) and healthcare investment (Alalawi et al. 2020). This conclusion has been derived both from the analytical rules in Table 5, which show that cooperation is favoured at lower densities in completely mixed populations and from the results obtained for networks of mixing communities with different topologies shown in Fig. 4. Low temperature, corresponding to limited mixing between communities, and smaller average group sizes consistently benefit the evolution of cooperation. This is in agreement with what has been observed in other approaches to the territorial raider model (Schimit et al. 2019), or even in the pairwise donation game in clustered networks (Allen et al. 2017).

Nonetheless, fixation probabilities sometimes display non-monotonic dependencies on home fidelity, raising the possibility that increased mixing between communities could, under certain spatial organisations, promote cooperation. Under mixing communities, interactions depend not only on the size and number of communities, but on how their home nodes are connected. We are thus interested in understanding in the future whether highly cooperative states may emerge under mixing communities, similarly to what has been found in parallel approaches to higher-order interactions in generalised versions of the multiplayer Prisoner’s Dilemma (Civilini et al. 2024), or considering dilemmas with non-rivalrous public goods and/or shared costs, such as the Snowdrift game, given their propensity to evolve cooperation under high group size variance (Archetti and Scheuring 2012; Santos et al. 2008; Gómez-Gardeñes et al. 2011b). This further raises the question of whether a unified measure could capture the combined effects of all these factors, a question that could be addressed by exploring a broader range of network structures with varying community sizes and home fidelity values.
